# A Simplified Method for Gene Knockout and Direct Screening of Recombinant Clones for Application in *Paenibacillus polymyxa*

**DOI:** 10.1371/journal.pone.0068092

**Published:** 2013-06-27

**Authors:** Seong-Bin Kim, Salme Timmusk

**Affiliations:** Department of Forest Mycology and Plant Pathology, Uppsala BioCenter, SLU, Uppsala, Sweden; Louisiana State University, United States of America

## Abstract

**Background:**

*Paenibacillus polymyxa* is a bacterium widely used in agriculture, industry, and environmental remediation because it has multiple functions including nitrogen fixation and produces various biologically active compounds. Among these compounds are the antibiotics polymyxins, and the bacterium is currently being reassessed for medical application. However, a lack of genetic tools for manipulation of *P. polymyxa* has limited our understanding of the biosynthesis of these compounds.

**Methods and Principal Findings:**

To facilitate an understanding of the genetic determinants of the bacterium, we have developed a system for marker exchange mutagenesis directly on competent cells of *P. polymyxa* under conditions where homologous recombination is enhanced by denaturation of the suicide plasmid DNA. To test this system, we targeted *P. polymyxa* α-and β-amylase genes for disruption. Chloramphenicol or erythromycin resistance genes were inserted into the suicide plasmid pGEM7Z-f+ (Promega). To mediate homologous recombination and replacement of the targeted genes with the antibiotic resistance genes nucleotide sequences of the α-and β-amylase genes were cloned into the plasmid flanking the antibiotic resistance genes.

**Conclusions:**

We have created a simple system for targeted gene deletion in *P. polymyxa E681*. We propose that *P. polymyxa* isogenic mutants could be developed using this system of marker exchange mutagenesis. α-and β-amylase genes provide a useful tool for direct recombinant screening in *P. polymyxa.*

## Introduction

*Paenibacillus polymyxa* (formerly *Bacillus polymyxa*), the type species of *Paenibacillus*, is considered to be a plant growth-promoting rhizobacterium (PGPR) with a broad host plant range [1]–[5]. *P. polymyxa* is widespread in the soil and is widely used in agriculture, industry, and environmental remediation because of its multiple functions [1], [2], [4]–[18]. Owing to its production of desiccation- and heat-resistant endospores, and to its intrinsic resistance to several fungicides and herbicides, it has high potential to be applied in agriculture as a biocontrol agent. The genomes of different strains of *P. polymyxa* have been sequenced by four groups [4], [19], [20] and Timmusk et al. (manuscript submitted for publication). The *P. polymyxa* E681 genome is composed of one circular chromosome of 5.4 Mb [19]. The genome of strain SC2 is composed of a circular 5 Mb chromosome and a 510 kb plasmid, and M1 is composed of 5.8 Mb chromosome and 360 kb plasmid [4]. The sequences reveal that *P. polymyxa* isolates have potential to produce various economically important biologically active compounds; however, a lack of genetic tools available for manipulation of *P. polymyxa* has hindered our understanding of the biosynthetic steps involved in natural product synthesis. Only two very important antibiotic compounds, fusaricidins and polymyxins, have been successfully mutagenized in the bacterium. This was performed by targeted mutagenesis protocol based on genomic libraries of PKB1 and E681 [21]–[23]. Direct homologous recombination between introduced DNA and *P. polymyxa* corresponding chromosomal loci has not been demonstrated previously probably due to low efficiency of transformation or to the low efficiency of homologous recombination events. Library-based mutagenesis is a costly and time-consuming approach and not realistic when a number of genes have to be mutated simultaneously. In this study α- and β-amylase genes were targeted for inactivation to test the mutagenesis system we developed.

The enzyme α-amylase randomly hydrolyzes α-1,4-linkages in both amylose and amylopectin chains. The enzyme β-amylase hydrolyzes alternate β-1,4 linkages from the ends of amylose and amylopectin chains. [24]. Although α-amylases are widely distributed in various kinds of living organisms, only certain plants and microorganisms are known to produce β-amylases [25]. Some microorganisms produce also glucoamylase which hydrolyzes α-1,3, α-1,4, and α-1,6 linkages. Because glucoamylase is less Efficient than α-amylase in cleaving α-1,4 linkages, it is usually used in conjunction with α-amylase [24].

Here we report on successful gene replacement in *P. polymyxa* E681 cells under conditions where homologous recombination is enhanced by denaturation of the suicide plasmid DNA. Alpha -and -beta-amylase genes were targeted to test the system. The genes provide a useful tool for direct recombinant screening in *P. polymyxa.*

## Materials and Methods

### 1. Bacterial Strains and Culture Condition

*Paenibacillus polymyxa* E681 was isolated from the rhizosphere of winter barley [26]. The primers for this experiment are described in Table 1. *Escherichia coli* DH5alpha cells (Invitrogen) acted as host for recombinant plasmids and were cultivated at 37°C on LB agar. *Bacillus subtilis* JH642 (Bacillus Genetic Stock Centre, BGSC; Ohio) was used for the complementation experiment. *B. subtilis* and *P. polymyxa* E681 were grown in half strength Tryptic soy broth (TSB) (Difco) at 30°C. Brain heart Infusion medium (Difco) containing 10% sucrose was used for the transformation of *P. polymyxa* E681. The extraction of plasmid and chromosomal DNA were performed according to standard procedures [27]. For antibiotic selection the media were supplemented with 5 µg/ml chloramphenicol, 1 µg/ml erythromycin and 100 µg/ml ampicillin, final concentration.

**Table 1:** Primers used in this study.

α-amyFF	5′-CTC ATG CAT GGA GTA GAC ATA GAG TCT GC-3′
α-amyFR	5′-CTC GGA TCC CCG TAG AAA TAC CCT TCG CC-3′
α-amyRF	5′-CTC TCT ACA AAG GCG GCA CTC GAT GCT CC-3′
α- amyRR	5′-CTC GGG CCC CTT CCC TGT GTG TAC TCT AA-3′
β-amy FF	5′-CTC ATG CAT CTT CTA CTA AGC CTT GTG CTG-3′
β-amy FR	5′-CTC GGA TCC AGG CTG CAT TAA TCT TGT TGT CC-3′
β-amy RF	5′-CTC TCT AGA AGA GCT GGT TGG TAA GGC AC-3′
β-amy RR	5′-CTC GGG CCC GCT TAG CGA TGT ACT GAT ACA G-3′
CatF	5′-AAAGGATCCTCATGTTTGACAGCTTATCATCG-3′.
CatR	5′-AAAGAATTCCCACGCCGAAACAAGCGCTC-3′.
EryF	5′-CTC ATG CAT GGA GTA GAC ATA GAG TCT GC-3′
EryR	5′-CTC GGA TCC CCG TAG AAA TAC CCT TCG CC-3′
C-α-amyF	5′-CTCCATATGGTCGACCCTGAACTGATTTGGACGAGAG-3′
C-α-amyR	5-CTCGGATCCGTTAACACCCCAAACTGGAT-3′
C-β-amyF	5′-CTCCATATGGAATTCACAGACTGGAACAAGGCTTC-3′
C-β-amyR	5′-CTCGGATCCCAGCCGACCGCTCTTCAAAG-3′

### 2. Construction of the Suicide Vectors

**Figure 1:**
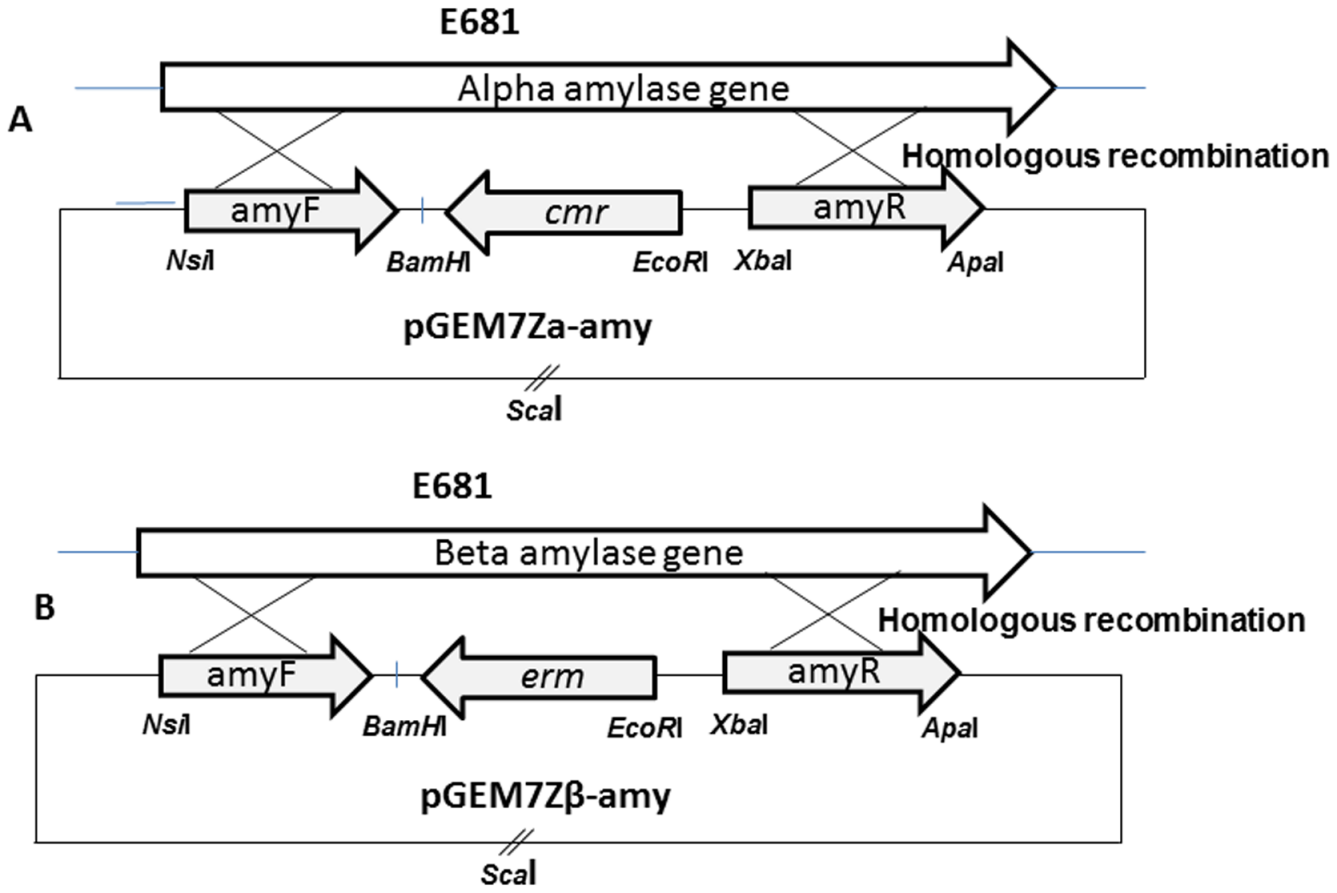
Schematic drawing of the construction of the shuttle plasmids pGEM7Zα-amy and pGEM7Zβ-amy.

A vector for targeted mutagenesis of *P. polymyxa* genes by replacement with a chloramphenicol or erythromycin resistance cassette was constructed from the plasmid pGEM7Z-f+ (Promega) which replicates in *E. coli* but not in *P. polymyxa* (Fig. 1).

#### Cloning strategy for the pGEM7Z α-amy vector for α-amylase gene replacement

The *cat* cassette encoding chloramphenicol resistance was constructed by PCR amplification using primers CatF and CatR from pDG1661 (BGSC, Ohio) and was then ligated into pGem7Zf(+) via EcoRI and BamHI cleavage sites resulting in plasmid pGEM7Zf Cat. Subsequently, 760 bp and 870 bp fragments of the flanking regions of the α-amy gene were amplified by PCR from the E681 chromosomal DNA using α-amy-FF – α-amy-FR and α-amy-RF – α-amy RR primer pairs resulting in PCR products A and B respectively. Fragments A and B were then introduced into pGem7Zf cat (+) using the NsiI and BamHI, and XbaI and ApaI restriction sites, respectively, resulting in plasmid pGEM7Za-amy.

#### Cloning strategy for the pGEM7Z β-amy vector for β-amylase gene replacement

The pGEM7zf Cat *cat* gene was replaced with the gene encoding erythromycin resistance (*erm*) using primers EmyF and EmyF. Then, 742 bp and 794 bp fragments of the flanking regions of β-amy gene were amplified by PCR from the E681 chromosomal DNA using β-amy –FF/β-amy -FR and β-amy –RF/β-amy RR primer pairs resulting in PCR products C and D respectively. The fragments were inserted to into pGem7Zf erm NsiI and BamHI, and XbaI and ApaI restriction sites resulting in plasmid pGEM7Zfb-amy.

### 3. Transformation Conditions for *P. polymyxa* E681

Transformation of *P. polymyxa* was performed by electroporation. In order to prepare competent cells, a single *P. polymyxa* E681 colony was picked from 1/2 TSA solid medium and cultured in BHIS broth [27] at 30°C and 200 rpm for 15 hours. Two ml of the pre-culture was inoculated into 200 mL of BHIS and cultured at 30°C, 200 rpm. When the cells reached an OD_600_ of 0.5, the bacterial growth was interrupted by placing the culture on ice for 10 min and centrifuged at 5000×*g* for 10 min at 4°C. After washing the cells twice with cold SM buffer [27], the competent cells were resuspended in SM buffer. Electroporation was performed using a Gene Pulser (Bio-Rad Laboratories, Richmond, Calif.).

It is known that homologous recombination may be strongly stimulated by denaturation of double-stranded circular DNA. DNA for electroporation was prepared after slightly modified protocols of Oh and Chater (1997) and Hinds et al (1999) [28]–[30] Briefly, DNA was denatured in a total volume of 20 ml containing 0.2 mM EDTA 0.2 M NaOH for 20 min at 37°C. Two µl of 3 M sodium acetate, pH 4.8, was added, and the mixture was purified using Qiaquick mini-columns (Qiagen). DNA was eluted with 18 ml of H_2_O, and kept on ice before electroporation. For UV treatment, 18 ml of DNA solution in a droplet on a plate was subjected to 2 50, 100 or 250 mJ cm^−2^ UV irradiation in a UV chamber (CL1000 UV crosslinker UVP Inc.), and DNA was kept on ice before electroporation. Competent cells were mixed with DNA, transferred to 2 mm cuvettes, and placed on ice for at least 5 min. The sample was pulsed with a voltage of 6.25 kV cm^−1^, a capacitance of 25 µF, and a resistance of 200 Ω. One ml of 30°C pre-warmed BHIS was added and the cell suspension was incubated at 30°C for 3 h.

Transformants were selected on respective antibiotics plates and cassette insertion was confirmed by PCR with primers flanking the sequences used in the mutagenesis vector.

### 4. Complementation in *Bacillus subtilis* JH642

As only partial complementation of amylase genes in *P. polymyxa* was achieved, the complementation was performed in *B. subtilis* JH642. For this purpose, the α-amylase gene of *B. subtilis* JH642 was first deleted by using pDG1662 integration plasmid (BGSC, Ohio). For the complementation of the α-amylase gene of *P. polymyxa* E681, the α-amylase gene was amplified with C-α-amyF and C-α-amyR then cloned into pHPS9 (BGSC, Ohio), an *E.coli*/Bacillus shuttle vector, via the NdeI and BamHI restriction sites. For complementation of the β-amylase gene, the full structural gene of β-amylase was amplified with C-β-amyF and C-β-amyR. This fragment was cloned into pHPS9 using the NdeI and BamHI restriction sites. These plasmids carrying amylase genes were introduced into *B. subtilis* by natural uptake. A fresh single colony cultivated overnight was inoculated in Spizizen’s medium [27] with 200 rpm at 37°C for 5.5 hours and then mixed with plasmid DNA for 1 hour; subsequently colonies were selected on LB agar containing chloramphenicol.

### 5. Measurement of Starch Concentration in Media

Production of α- and β- amylase was quantified as follows. Each of the strains was inoculated in half strength tryptic soy broth (TSB) containing 1% soluble starch (Sigma-Aldrich, Cat# S2004). An aliquot of the culture was removed at each measurement time and centrifuged at 8,000×g for 10 min at 4°C. The supernatant was diluted ten times with distilled water and 150 µl of diluted supernatant was added to 600 µl of reaction buffer (50 mM Tris Cl pH 6.8, 25 mM CaCl_2_ 2H_2_O), followed by 300 µl of detection solution (0.01% w/v I_2_ and 0.1% w/v KI in 1 M HCl). The starch concentration was measured by changes in the optical density at 620 nm.

## Results and Discussion

There is considerable interest in *P. polymyxa* because this bacterium produces various biologically active compounds [1], [2], [21], [31], [32]. However, the lack of an efficient direct gene manipulation system for this organism has hampered further genetic studies. Genome analysis depends largely on the ability to assign functions to the various putative ORFs identified by DNA sequencing. For a given gene, the most straightforward approach is to create a knockout in that gene and to characterize the phenotype of the mutant. In order to verify the roles of *P. polymyxa* genes *fusA* and *pmxE* in fusaricidin and polymyxin biosynthesis, respectively, their genome library based targeted mutations were created [21]–[23]. Construction of a vector for mutagenesis was based on the lambda red recombinase system developed by Datsenko and Wanner for use in *E. coli* and adapted for mutation in many other bacteria [33], [34]. *P. polymyxa* genes were inserted into a cosmid vector and introduced into *E. coli* for insertion of a PCR-amplified antibiotic resistance cassette to target the *P. polymyxa* gene by homologous recombination. Both conjugation and electroporation procedures were used to introduce the altered vector in to *P. polymyxa* for gene disruption [14], [21]. In contrast to these methods, our gene knockout vectors were inserted directly into E681 competent cells. Previous attempts to carry out gene targeting by homologous recombination directly into *P. polymyxa* have been unsuccessful. We have applied a method used in mycobacteria and *Streptomyces spp*. [29], [30] This methodology exploits the organism’s endogenous machinery of DNA repair and/or recombination to integrate DNA at a target locus as directed by DNA sequence homology. UV-irradiated or alkali-denatured DNA molecules may provide a more recombinogenic substrate for DNA repair and/or recombination systems of the bacterial cell. Introduction of altered DNA into the bacterial cells by electroporation resulted in 20 to 100 transformants per µg DNA from which 10 to 60% were allelic exchange colonies. The stimulatory effect of homologous recombination was maximum after DNA UV irradiation at 50 mJ cm^−2^.The mutated bacterial colonies exclusively carried flat phase variation. Phase variation is an adaptive process by which bacteria undergo frequent and reversible phenotypic changes resulting from genetic alterations at specific loci of their genomes. This process is known to be crucial for the survival of various pathogens and facilitates adaptation of bacterial populations to stressful environments [35]–[37]. Phase variation in *P. polymyxa* is revealed as two different colony morphologies: bold opaque colonies (B type) and flat colonies (F type). Similar colony morphology has been observed e.g. in *P. aeruginosa*. The mechanism of phase variation includes site specific recombination, gene conversion, epigenetic modifications, nested DNA inversions and slipped strand mispairings but could also involve recently reported new mechanisms [36], [38]. Introduction of altered DNA into the bacterial cell may recruit the recombination machinery for initiating homologous pairing and strand exchange. The stimulation of homologous recombination by UV may be caused by the production of a gapped duplex substrate following nucleotide excision repair (by *UvrABC).* This has been shown to be an effective recombination substrate owing to the rapid formation of *RecA* filaments [28] It is likely that such a genetic arrangements can lead to F colony type preference in E681 during the mutagenesis. The direct mechanism of *P. polymyxa* phase variation remains to be elucidated. Yet it is already clear that on and off switching of phenotype expression is involved when manipulating the bacterial populations.

**Figure 2:**
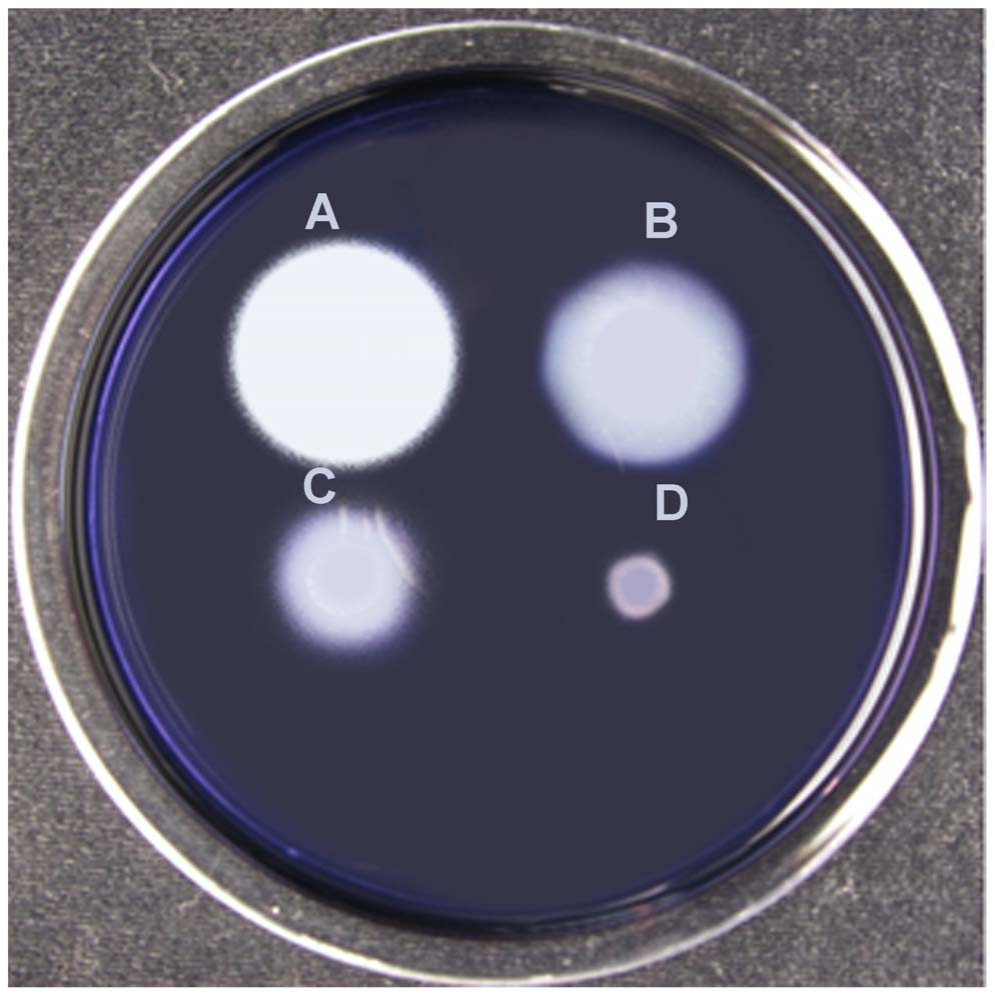
Starch clearance plate assay to detect amylolytic activity of (A). *P. polymyxa* E681 (B). *P. polymyxa* E681, Δ α-amy:: *cat* **(C).** *P. polymyxa* E681, Δ β-amy::*erm* (D). *P. polymyxa* E681, Δ α-amy:: *cat*, Δ β-amy::*erm* **.** Note that (**D**). *P. polymyxa* E681, Δ α-amy:: *cat*, Δ β-amy::*erm* has lost the amylolytic activity indicating that both genes are required for starch degradation.

In order to demonstrate the functionality of this gene knockout system, deletion mutations of *P. polymyxa E681* α*-*amylase and β*-*amylase genes PPE_02348 and PPE_04705 were created. In addition to testing the system the genes encode enzymes that participate in starch degradation and hence provide a system for direct screening for various heterologous fragments to the genes to *P. polymyxa* on starch clearing plates. For deletion of the *α -*amylase gene, its structural gene flanking regions were cloned into a pGEM7Zderivative containing the *cat* gene which inactivates chloramphenicol. For the β*-*amylase gene deletion the gene flanking regions were cloned into a pGEM7Z derivative containing the *erm* gene which encodes erythromycin resistance (Fig. 1A and B). After transformation, it was found that the α-amylase mutant has reduced amylolytic activity forming an almost identical starch clearance zone as the wild type (Fig. 2). This is in contrast to the results obtained with the β-amylase gene which generated a clearly reduced zone of clearance on starch plates (Fig. 2). Double mutants show full inhibition of starch degradation on the plate assay confirming that both genes are required for starch degradation in *P. polymyxa* (Fig. 2). Our results clearly show that upon insertion of heterologous fragments (in our case *cat* and *erm* genes) into the α-and β-amylase genes their amylolytic activity was disrupted. β-amylase and double mutant show distinct differences in starch clearance on plate assays. These phenotypes therefore allow the direct screening of recombinant clones. Further, the identification of the recombinant clones can be performed directly on the transformation plates by a simple assay. In contrast to other screening possibilities commonly employed for positive differentiation of recombinants, starch clearance plates provide excellent results at modest costs.

**Figure 3:**
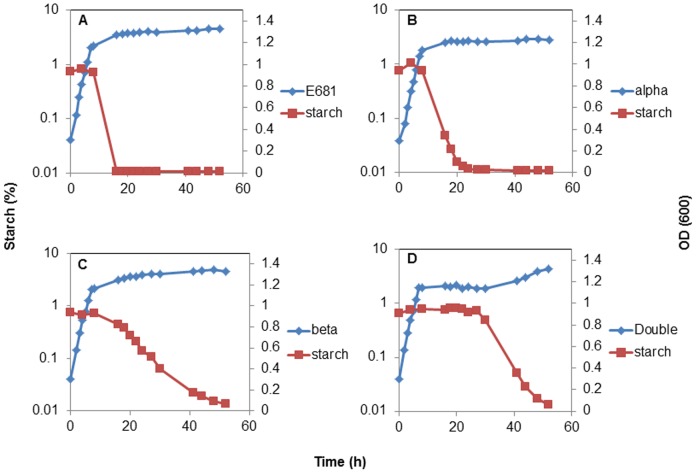
Starch degradation profiles in liquid assays of (A). *P. polymyxa* E681 (B). *P. polymyxa* E681, Δ α-amy:: *cat* (alpha) (C). *P. polymyxa* E681, Δ β-amy::*erm* (beta) (D). *P. polymyxa* E681, Δ α-amy:: *cat*, Δ β-amy::*erm* (double). Note that amylolytic activity of beta amylase mutant is lost during the 20 h of growth and 32 h in case of double mutant (D) in ½ TSB medium.

**Figure 4:**
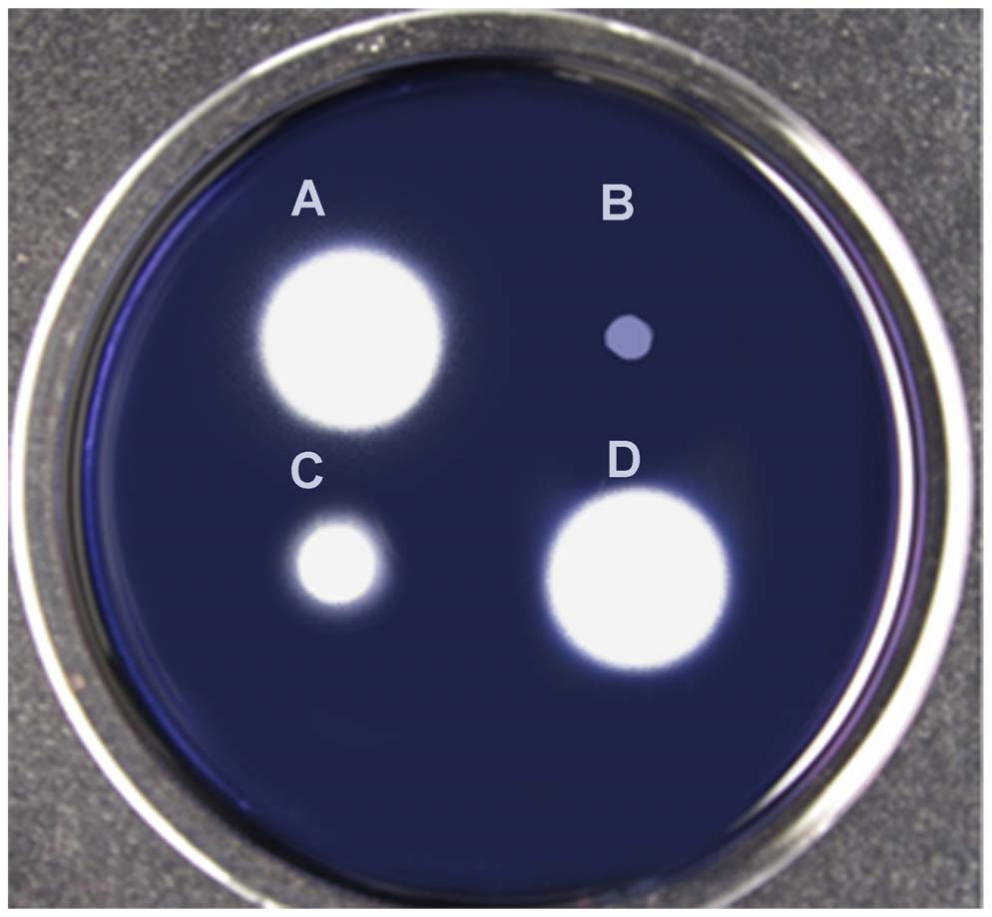
Starch clearance plate assay to detect complementation of amylolytic activity of (A). *B. subtilis* JH642 (B). *B. subtilis* JH642, Δ α-amy:: *cat* (C). *B. subtilis* JH642, Δ α-amy:: *cat,* pHPS9-α-amylase gene (D). *B. subtilis* JH642, Δ α-amy:: *cat,* pHPS9- β-amylase gene. Note that α- and β- amylase complementation restores full amylase production (D).

To confirm disruption of the amylase genes, we studied the dynamics of *P. polymyxa* E681 starch degradation in ½ TSB over 60 h (Fig. 3A–D). The results confirm that amylolytic activity is only initiated at the beginning of stationary phase of bacterial growth and that α- and β-amylase deletions do not influence bacterial growth in TSB medium. The liquid assay results agree with the starch clearance plate assays results for the first 20 h. After that time β-amylase mutant starch degradation ability increases, rapidly depleting the starch by 50 h. Double mutant starch degradation ability remain low for over 32 hours after which it rapidly increases and forms a pattern nearly identical to the wild-type and the α- and β-amylase mutants after 50 h. These results suggest that enzymes other than α- and β-amylase are involved in starch degradation of *P. polymyxa* during late stationary phase. The complementation of amylase genes was attempted in *P. polymyxa* E681derivatives using pHPS9, an *E.coli/Bacillus* shuttle vector. The α- amylase gene was amplified with C-α-amyF and C-α-amyR primers and β- amylase with C-β-amyF and C-β-amyR primers. The amplified products were inserted into pHPS9 between NdeI and BamHI restriction sites. Yet only 25% starch degradation with complementation experiment was achieved. It is likely that the p59 promoter driving the expression of cloned amylase genes in pHPS9 had a reduced activity in *P. polymyxa*. Hence, the complementation experiment with amylase genes was performed in *B. subtilis* JH642. Initially the JH642 α-amylase gene was deleted and replaced by *P. polymyxa* α- and β-amylase genes (Fig. 4). However, only insertion of both genes restored the full starch degradation pattern of JH642 (Fig. 4 C and D).

While the function of many genes in the *P. polymyxa* genome may be inferred from sequence similarities, the functions of the majority of the genes are unknown. In this study, we developed a new genetic tool for *P. polymyxa* that enables efficient and less costly creation of gene knockouts. To our knowledge, this represents the first reported use of gene replacement by homologous recombination based on *P. polymyxa* cells. Likewise we show that the α- and β-amylase genes provide a valuable genetic marker that enables rapid direct screening of heterologous genes in *P. polymyxa* cells. Even though this study focused on the specific insertional mutagenesis of *α* - and *β-*amylase genes, the technique described could be used more generally for mutagenesis of various genes in *P. polymyxa.* The isolation of mutants deficient in important functions would be extremely useful in elucidating the genes that contribute to the beneficial effect of *P. polymyxa.*
